# Effect of Moisture on Powder Flow Properties of Theophylline

**DOI:** 10.3390/pharmaceutics2030275

**Published:** 2010-07-02

**Authors:** Niklas Sandler, Katharina Reiche, Jyrki Heinämäki, Jouko Yliruusi

**Affiliations:** 1 Pharmaceutical Sciences, Department of Biosciences, Åbo Akademi University, Turku, Finland; 2 Division of Pharmaceutical Technology, Faculty of Pharmacy, University of Helsinki, Finland

**Keywords:** powder flow, relative humidity, equilibrium moisture content, solid-state properties, theophylline

## Abstract

Powder flow is influenced by environmental factors, such as moisture and static electricity, as well as powder related factors, such as morphology, size, size distribution, density, and surface area. Pharmaceutical solids may be exposed to water during storage in an atmosphere containing water vapor, or in a dosage form consisting of materials (e.g., excipients) that contain water and are capable of transferring in to other ingredients. The effect of moisture on powder flowability depends on the amount of water and its distribution. The aim of this work was to examine the effect of humidity on the flow properties of theophylline using information derived from solid-state analysis of the systems investigated.

## 1. Introduction

The process design of tablets, inhalation powders, granules, pellets, and capsules strongly depends on the flowability properties of each individual powder. The flowability is of critical importance when concerning uniform feed from bulk storage containers or hoppers into the feed mechanism of tabletting or capsule-filling equipment. Proper flow allows uniform particle packing and a constant volume-to-mass ratio, which maintains tablet weight uniformity. Additionally, only a good flowability can guarantee the reproducible filling of tablet dies and capsule dosators; good flowability improves weight uniformity and allows tablets to be produced with more consistent physicomechanical properties. Uneven powder flow can result in excess entrapped air within powders, which in some high-speed tabletting conditions may promote capping or lamination [1].

### 1.1. Mechanisms behind powder flow

A powder is flowing when the mobilizing gravitational forces are higher than particle-particle-interaction forces. According to Dawoodbhai *et al.* [2], the two fundamental particle-particle-interaction forces are cohesion and friction. Friction acts as a tangent to the contact point surface and opposes the relative motion of a particle. It increases with the true contact area and may decrease if moisture acts as a lubricant. Cohesion is the mutual attraction between two particles, which acts perpendicularly to the tangent of the contact point surface. It is defined as the resistance to separation of contacting powder particles of identical material. The contact between surfaces of the same material can also be named autoadhesion [3]. The total force of adhesion is known to be a summation value of several additive force components [4].

F_ad_ = F_vdw_ + F_c_ + F_e_ + F_es_

where F_ad_ is the force of adhesion, F_vdw_ is the Lifshitz-van der Waals’ force, F_c_ is the capillary force, F_e_ is the electrical force, and F_es_ is the electrostatic (Coulomb) force [3,4]. Dawoodbhai *et al.* [2] propose to include solid bridges to the cohesive forces. The van der Waals’ force is proportional to the particle size. But as the number of particle contacts per square unit is indirectly proportional to the square of particle size, the smaller the particles are, the higher the sum of the van der Waals’ forces are [5]. The capillary force or liquid bridges depends on the percent of water and its distribution. Contributing factors are the interfacial tension and capillary pressure [2]. The capillary force has been identified as the force which dominates over any other force component, once the relative humidity of the air has reached a value of 65 to 75% [6,7,8]. Capillary forces are inversely proportional to the particles’ size, but the effect described above is also valid for these forces. Consequently, in a bulk of smaller powder particles, higher capillary forces are acting [5]. The value of F_e_ is a constant for two materials in contact, and F_e_ cannot arise if moisture is present in the gap between the contiguous surfaces [8]. An electrostatic force, F_es_, only participates if the particles and/or surfaces have been actively charged [3,9,10].

Powder flow is influenced by environmental factors, such as moisture and static electricity, as well as powder related factors, such as morphology, size, size distribution, true density, surface area [11], and chemical composition [5]. Additionally, the previous history of the material, which means the previous consolidation stress, can have an influence on powder flow ability [12].

### 1.2. Moisture and powder flow

Pharmaceutical solids may be exposed to water during storage in an atmosphere containing water vapor, or in a dosage form consisting of materials that contain water (e.g., excipients) and are capable of transferring in to other ingredients [13]. The effect of moisture on the flowability depends on the amount of water and its distribution [2]. Water can interact only at the surface of solids (adsorption), or water can penetrate the bulk solid structure (absorption) [14]. The amount of water sorbed is a function of the affinity between the surface and the water molecules, temperature, relative humidity (RH), and in case of adsorption, and also the amount of exposed surface area [15,16]. If the water is absorbed, it can lead to a physicochemical change of the powder material, e.g., formation of a hydrate, such as with anhydrous theophylline. But the humidity can also simply be integrated into the powder particle, which can be seen for lignite [5]. If water is adsorbed onto the surface of the powder particles, the water distribution will depend on the hydrophilic properties of the powder [17].

Airaksinen [15] reviewed the interactions between moistures and solids. The adsorption process occurs with the water forming hydrogen bonds with the hydrophilic sites on the surface of the solid [18]. Water molecules first adsorb onto the surfaces of dry material to form a monomolecular layer, which is subjected to both surface binding and diffusional forces. Diffusional forces exceed the binding forces as more water molecules adhere to the surfaces and moisture is transferred into the material [19]. Thus, moisture is adsorbed as mono- or multilayers or may be present as normally condensed moisture. Multilayer water adsorption consists of water uptake into pores and capillary spaces, dissolution of solutes, and finally the mechanical detention of water [20]. 

Important measurement categories concerning moisture are the relative humidity of the air, the water activity, and the equilibrium moisture content of a powder sample. The relative humidity of an air-water mixture is defined as the ratio of the partial pressure of water vapor in the mixture to the saturated vapor pressure of water at a prescribed temperature. The water activity (a_w_) or equilibrium relative humidity (ERH) is defined as the ratio of the water vapor pressure of the substance to the vapor pressure of pure water at the same temperature [15]. The moisture content at which a solid material produces a water vapor pressure equal to that of the surrounding environment is defined as the equilibrium moisture content (EMC) [2].

Recently, humidity dependent changes in flowability have been studied by Emery *et al.* [21]. Changes in technological properties, such as flowability, depend on the water amount and on the distribution of water molecules in the solid. Lower amounts of water might have a positive effect on powder flow, as it can eliminate particle micro-irregularities and electrostatic charges [22]. A higher amount of water will increase the thickness of the adsorbed liquid layer, which increases the strength of liquid bridges [21]. Consequently, the powder becomes more cohesive and tends to form agglomerates [22]. Most studies observe a decrease in powder flow with increasing moisture [21].

### 1.3. Powder flow measurement

The primary intent of flow measurements is the characterization of bulk solid flow properties, but often, the measured data are used to design equipment for storage, transportation, or general handling of bulk solids. Flowability testing is also needed to compare the flowability of similar or competing bulk solids, to determine whether a product fulfills the requirement of quality control, to model processes with the finite element method, or to judge any other process in which the strength or flowability of bulk solids plays an important role [23]. Powder flow measurement techniques are many and descriptions of these can be found elsewhere [1,23]. One of the simplest methods of determining powder flowability directly is to measure the rate at which powder discharges from a hopper. By dividing the discharged powder mass by the discharging time, a flow rate is obtained, which can be used for quantitative comparison of different powders [1]. Apart from shear cell methods, commonly used devices fail to differentiate between poorly flowing materials. As an extension to the above mentioned flow rate measurement technique, a new technology has been developed, which combines a mechanical up- and down oscillation with the recording flow meter, and enables very small samples sizes (*ca.* 1g) [24]. The device has also been shown to enable the measurement and differentiation between small differences in poorly flowing cohesive powders. For this reason, in this study, this method is used in powder flow characterization. The aim of the present work was to examine the effect of humidity on the powder flow properties of theophylline. Also, the applicability of a novel flow measurement technique is exploited in the measurements of these cohesive powder systems. The data interpretation is supported through solid-state analysis of these systems.

## 2. Experimental Section

### 2.1. Materials

To obtain a sample that is, with a high degree of assurance, theophylline anhydrate, the theophylline anhydrate bulk material, 200 M (Orion Pharma, Espoo, Finland) was dried at 100 ºC for 4 days. For reference purposes, pure theophylline monohydrate was produced by recrystallization of theophylline anhydrate bulk material (200 M in water at a temperature of 65 ºC). 

### 2.2. Storage conditions of theophylline

Altogether, 34 g of anhydrous theophylline bulk material, 200 M (Orion Pharma, Espoo, Finland) were filled into open plastic containers and were each conditioned at 5 different humidities and 300 mbar for nine days. Constant humidities were achieved with desiccators, which were filled with different over-saturated salt solutions, water, or silica beds. The different contents in the dessicator were pure water (RH = 99%), NaCl (RH = 82%), NaBr (RH = 63%), MgCl (RH = 39%), and dried silica beds (RH ≤ 19%). The humidity was monitored over the conditioning time with Thermo-Hygrometers (Widder, Germany, accuracy of humidity: ±5% RH at 25% up to 75% RH). 

### 2.3. Sample analysis

The samples, which were conditioned at different humidities for 9 days, were weighted immediately after opening the desiccator. Afterwards, the water activity was determined. After every operation step, the desiccator was closed again, to keep the samples as close as possible to their specified humidity. For every flow measurement, the powder was directly taken out of the desiccator, and sieved through a 297 µm sieve, by forcing it through the sieve with a card. The powder was then filled as fast as possible into the hopper of the FlowPro (powder flow analyzer). The rest of the powder from these sieving procedures was used for the Bulk- and Tapped Density experiments. The angle of repose was determined out of the cone that was formed from the powder flowing out of the FlowPro hopper. Immediately after these flow measurements, the powder was analyzed with the moisture analyzer, the Raman spectrometer, the X-ray powder diffractometer, and the Laser-diffractometer. Scanning electron microscope analysis was also carried out.

### 2.4. Amount water in the system

#### 2.4.1. Weight difference

Before and after storage in the desiccator, the samples were weighted with a precision balance (Mettler Toledo, AX105 Delta Range, Switzerland). Measurements were made four times each. A relative mass difference was calculated using Excel 2003 (Microsoft). 

#### 2.4.2. Moisture analyzer

The water content of the samples was analyzed using a Moisture Analyzer (MA100, Sartorius AG, Goettingen, Germany) with a heating-cycle of up to 105 ºC. Measurements were made once. 

#### 2.4.3. Surface water (Water activity)

The vapor pressure of water above the samples was determined using an AquaLab, Model Series 3TE (Decagon Devices, Inc., Washington, USA). Measurements were made once. 

### 2.5. Solid –state analysis

The phase transition from theophylline anhydrate to theophylline monohydrate was observed by Raman spectroscopy and XRPD (X-Ray Powder Diffraction). 

#### 2.5.1. Raman spectroscopy

Raman spectroscopy was performed with the P^h^ AT System ^TM^ (KAISER optical systems, INC, USA), consisting of a baseunit with a camera, spectrograph, and Laser (785 nm, Invictus) and a Probehead. Measurements were carried out using HoloGRAM-Software (3 seconds, 3 acums, auto new dark and cosmic ray filter). Measurements were made once. The data was converted into txt-file using a HoloGRAMs utility. The txt-files were converted to graphics using SigmaPlot 2000. 

#### 2.5.2. XRPD

X-Ray diffraction patterns were measured using an X-ray powder diffractometer (D8 Advance; Bruker axs, Germany). The source of radiation was a KRISTALLOFLEX 760 X-ray generator (Siemens, Germany). The measuring range was 2θ = 5°–40°, with a step size of 0.05º and a time per step of 1 s. Measurements were made once. The data was evaluated using EVA (DIFFRAC^plus^ Evaluation Program).

### 2.6. Size and surface of the particles

#### 2.6.1. SEM: Scanning electron microscopy

The steel-sample holder was prepared with a double-faced adhesive tape (Nisshin em. Co. ltd). The powder was distributed on the sticky upper side and non-sticking powder particles were removed with clean air spray (Dust off, Falcon, USA). The platinum-coating was carried out with the Agar sputter coater (Agar scientific) under vacuum and an Argon atmosphere (≈ 0.08 mbar). Pictures were taken with a DSM 962 scanning electron microscope (Zeiss, Germany)

#### 2.6.2. Laser diffraction

The volume particle size distribution was determined using a method based on laser light diffraction (Laser Diffraction Particle Size Analyzer LS13 320, Beckman Coulter, Inc., Miami, FL, USA). The samples were dispersed and measured in isopropanol trifold. 

### 2.7. Flow properties

#### 2.7.1. Flow Pro

The flow rate was determined using the FlowPro (SAY Group Oy, Finland). This flow meter measures the mass of a powder that flows through a hopper assisted by vertical oscillations, which break the cohesive forces [24]. The procedure is as follows: Sieved powder was filled into the steel hopper of the FlowPro in its loose-dilated state. The steel hopper has an inclination angle of 67º, an inner volume of 4.69 mL, and the orifice has a diameter of 3 mm. After filling, the hopper was fixed to the mounting and the operation was started by the software (FlowPro II). The up- and down-movements have a frequency of 1 Hz. The movements are such that the sample holder remains in the “down” position for 80% of the cycle, and in the “up” position for the remaining 20%. The flow rate [mg/s] was determined by measuring the mass over the time. To avoid irregularities at the beginning and at the end of the procedure, the first 10% mass change and the last 20% mass change were cut away. Measurements were made in triplicate.

#### 2.7.2. Angle of repose

The cone, which formed from the powder flowing out of the FlowPro, was photographed three times and analyzed with ImageJ software.

#### 2.7.3. Bulk and tapped density

Powder was filled into a 25 mL graduated cylinder. The mass was measured and the volume of the powder determined before and after 1250 taps. Measurements were made three times. 

### 2.8. Data evaluation

Anova-Analysis was performed with Excel 2003 (Microsoft). Tukey comparisons were made with Systat, Version 5.0 (Systat Software, Inc.). The null-hypothesis was rejected with a level of significance (P-value) of less than 5%.

## 3. Results and Discussion

### 3.1. Water in the systems

**Figure 1:**
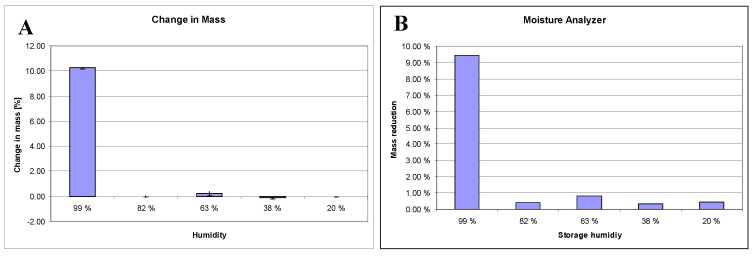
Effect of humidity conditions (RH) on the moisture content of theophylline samples determined by **(A)** a gravimetric method (n = 4) and **(B)** a moisture analyzer with a heating-cycle of up to 105 ºC (weight loss).

The mass change of the powder after storage in the desiccator is presented in Figure 1A. The weight loss of the conditioned powders, resulting from heating up to 105 ºC, is shown in Figure 1B. The weight differences between the untreated and the conditioned powder, as well as moisture, analysis, reveal a phase transition from theopylline anhydrate to theophylline monohydrate for the 99% RH conditioned sample only. The weight gain between theophylline anhydrate and the conditioned powder at 99% RH was 10.2%. The theoretical difference for a complete phase transition is 10.0%. Weight loss, which was measured using the moisture analyzer, was 9.4%. The theoretical weight loss for a complete phase transition is 9.1%. Both methods indicate a complete phase transition from theophylline anhydrate to theophylline monohydrate at 99% RH. For lower humidity, the mass did not significantly change. Therefore, a transition to the monohydrate at lower humidity did probably not take place. The mass reduction at lower humidity measured using the moisture analyzer was between 0.3% and 0.8%. This water seems to be free water, which forms layers around the powder, rather than crystal water. Raman and X-ray analysis confirmed this hypothesis. 

**Figure 2:**
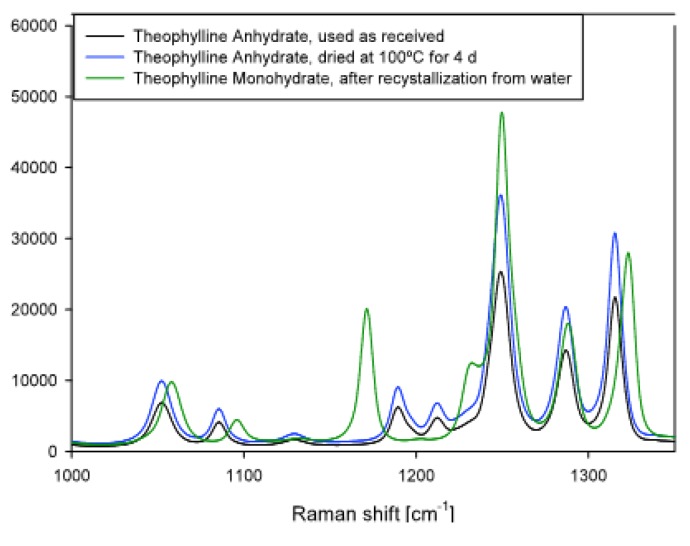
Raman spectra of theophylline anhydrate and monohydrate. Theophylline monohydrate shows a specific peak at 1171 cm^-1^, which does not exist for theophylline anhydrate. Both theophylline anhydrate samples show the same Raman spectrum.

**Figure 3:**
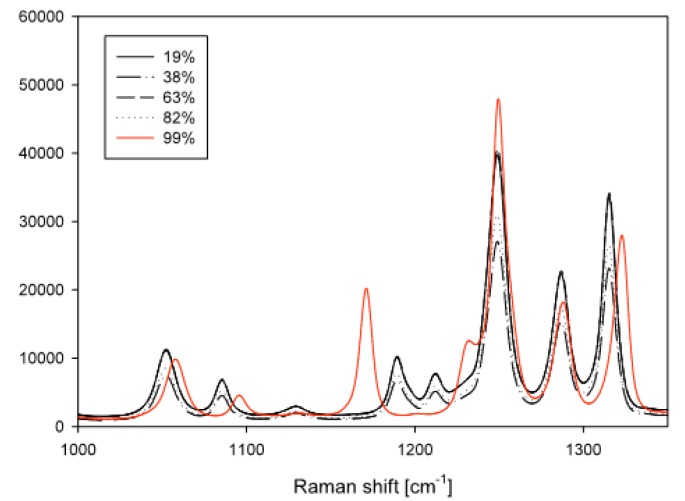
Raman spectra of theophylline anhydrate samples conditioned at 19%, 38%, 63%, and 82% RH, and at 300 mbar for nine days. The Raman spectra show the same spectra as the anhydrate reference. The sample conditioned at 99% RH shows the specific theophylline monohydrate peak at 1171 cm^-1^.

The reference samples for crystal structure analysis prepared according to Section 2.2 were observed with Raman spectroscopy. Spectra are shown in Figure 2. The Raman spectra of the conditioned powders are presented in Figure 3. The theophylline samples, which were conditioned at 19%, 38%, 63%, and 82% RH, show the same spectra as the anhydrate reference sample. The 99% RH conditioned sample shows the specific theophylline monohydrate peak at 1171 cm^-1^. This peak could not be observed for theophylline powders stored at 82%, 63%, 38%, or 20% RH. Consequently, a change of the crystal structure from the anhydrate to the monohydrate takes place only at 99% RH. The diffraction pattern of the powder that was conditioned at 99% RH shows the same interference maxima as the theophylline monohydrate. All the other conditioned samples have the same diffraction patterns that theophylline anhydrate has. Such observations further indicate a theophylline anhydrate phase transition to theophylline monohydrate at 99% RH, but not at lower humidities. 

This phenomenon has also been observed by Trask *et al.* [25]. A phase transition took place at 100% RH, but did not occur at 75% (oversaturated NaCl-solution) over a period of seven weeks. This strong borderline between a complete transition after a short time at 99% RH and no transition at slightly lower humidities over a long period can be explained with critical relative humidity, RH_0_ [17,25]. According to the theory, dissolution of water-soluble crystalline substances does not occur below RH_0_. Typically, poor water-soluble substances, like theophylline, have RH_0_ values around 90% at 25 ºC [17]. Consequently, at lower humidities, water vapor already condenses at the surface of the particle, but the amount of water is not sufficient to dissolve the particle. Without dissolution, no solution-mediated phase transition can take place. Only the theophylline stored at 99% RH showed caking, a clear sign for temporary dissolution. 

The angle of powder X-ray interference maxima for the reference samples and the conditioned samples are presented in Table 1. For clarity reasons, only the peaks between 2θ = 5º–15º are presented. Both theophylline anhydrate blank samples show the same diffraction pattern. The theopylline anhydrate shows a different interference maximum from that of the theophylline monohydrate blank. Theophylline, which has been stored at 99% RH, shows the diffraction pattern of theophylline monohydrate. The powders which have been stored at 82% RH or lower humidities show the same interference maxima as theophylline anhydrate. 

**Table 1:** Table presenting the typical XRPD peak maxima for the samples studied in the 2θ = 5º–15º range. T = theophylline.

T.Anhydrate, used as received	T.Anhydrate, dried at 100 ºC	T. Monohydrate	99%	82%	63%	38%	19%
	7.2			7.2	7.2	7.2	7.2
		8.85	8.85				
		10.4	10.4				
11.5	11.5	11.5	11.5	11.5	11.5	11.5	11.5
		12	12				
12.8	12.7			12.7	12.7	12.7	12.7
		13.4	13.4				
14.5	14.5			14.5	14.5	14.5	14.5
		14.7	14.7				

### 3.2. Surface water

**Figure 4:**
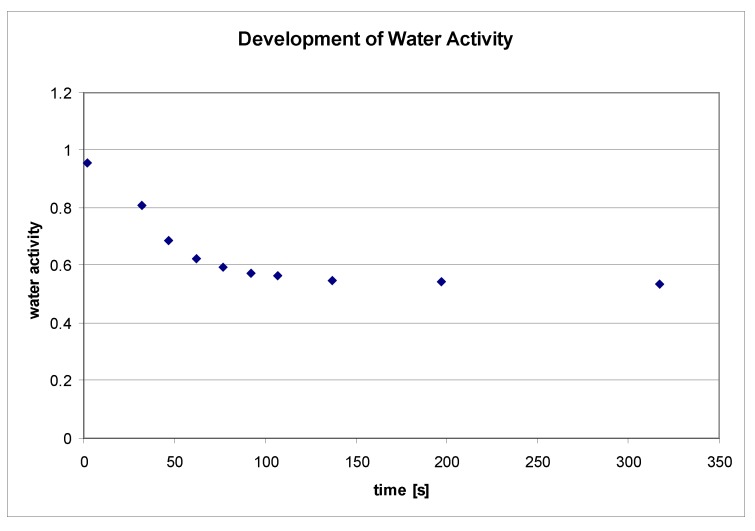
Water activity of a theophylline powder sample as a function of time. Theophylline was pre-conditioned at 99% RH and 300 mbar for nine days.

The water activity of an empty sample holder was found to be the same as the relative humidity of the circumfluent air. The water activity of a dried powder was also found to be the same as the relative humidity of the circumfluent air. The water activity of water was found to be 0.999. In Figure 4, the water activity of a theophylline powder, which had been conditioned at 99% RH, is plotted against time. After two minutes, the powder surface had almost completely acclimated to the humidity of the room with all samples, showing that the water activity was found to be very close to the relative humidity of the circumfluent air (0.514). Initial water activities of all conditioned samples (99%, 82%, 63%, 38%, and 20% RH) were 0.971, 0.518, 0.266, 0.460, and 0.409, respectively. After two minutes, the water vapor over a theophylline monohydrate sample, removed directly from the 99% RH dessicator to the measurement device, had changed to the humidity of the room. This development represents the water activity at the surface of the sample. Probably inside the bulk, the humidity will stay over a longer period. Interestingly, these measurements show how delicate the surface properties of materials are when concerning the relative humidity of the working atmosphere and the time for preparation and performance of the experiments. The water activities of the conditioned samples are not consistent with the humidity of the desiccators in which they were stored. It is likely that they had already been adapted to the humidity of the circumfluent air. 

### 3.3. Size and surface of the particles

The particles of the powder, which had been conditioned at 19%, 38%, 63%, and 82% RH all looked the same. Only the 99% RH conditioned theophylline powder had different particle surface properties upon visual inspection through the SEM micrographs. The particles of the lower humidity groups are composed of layers or crystal steps, as can be seen in Figure 6E. These layer formations are shown in another dimension in Figure 6F. These particles appear rougher. A closer look at the SEM figure 7B shows that the 99% RH sample, which has converted to the monohydrate form, reveals a change in the surface; the layers are not strictly separated anymore, but form a continuum. Not a single structure with discrete layers or a crystal step structure could be found. Additionally, the smaller particles are not agglomerated to the surface anymore (e.g., Figure 6F), but are coalesced with the smooth surface of the bigger particles. The particle size distribution, measured by laser diffraction, is the same for all samples indicating a mean particle size diameter (d50) between 41.5–47.2 µm for all samples. Differences of particle sizes between the different conditions are not statistically significant (ANOVA, P-value = 0.186). The data from laser diffraction should be regarded with some criticality, as the solvent might have an effect on the size of the particles. Additionally, a settling down of heavy particles was not fully avoidable. However, SEM analysis also shows that particle size and size distribution stay about the same after phase transition, but the surface of the particles changes. Temporary dissolution is the most probable explanation for the surface alteration. This experiment underlines the statement of Rodríguez-Hornedo *et al.* [26], that this phase transition is a solvent-mediated process.

**Figure 5:**
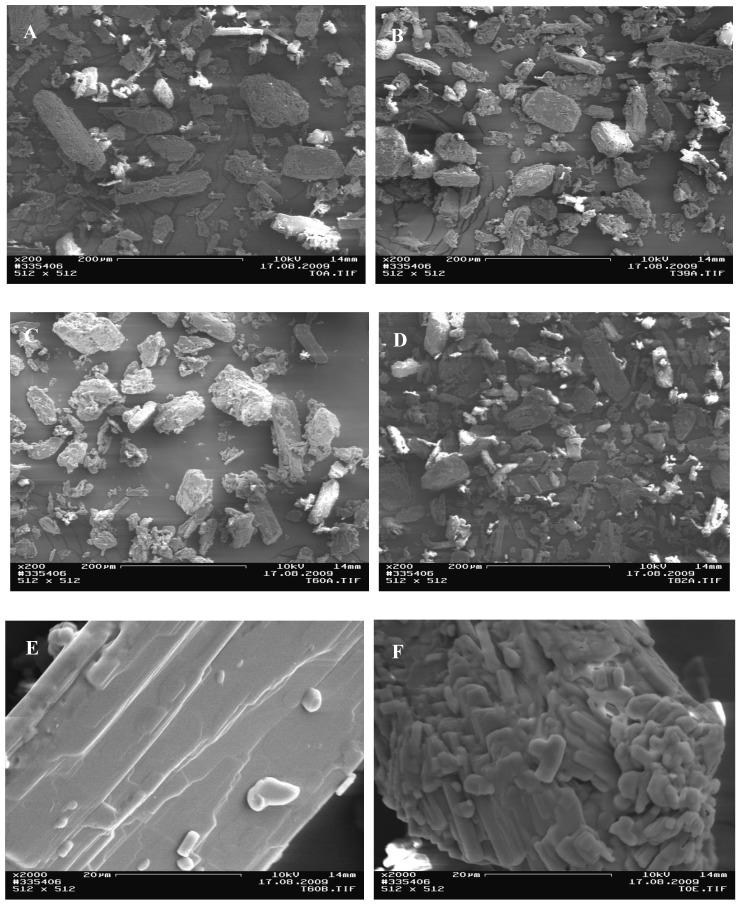
Scanning electron micrographs (SEMs) of theophylline samples conditioned at 19% RH **(A,E)**, 38% RH **(B)**, 63% RH **(C)**, and 82% RH **(D,F)**.

**Figure 6:**
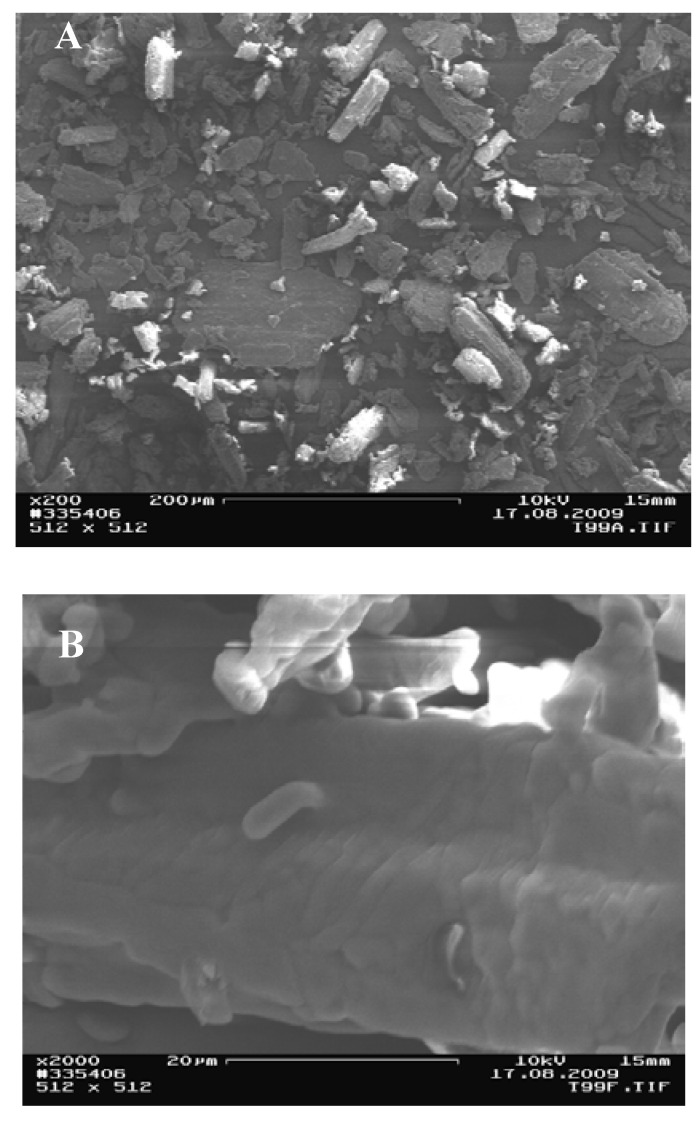
Scanning electron micrographs (SEMs) of the theophylline sample conditioned at 99% RH, at 200 × **(A)** and 2000 × **(B)** magnification 200. The smaller particles are not agglomerated to the surface anymore, but are coalesced with the smooth surface of the bigger particles.

### 3.4. Flow properties

**Figure 7:**
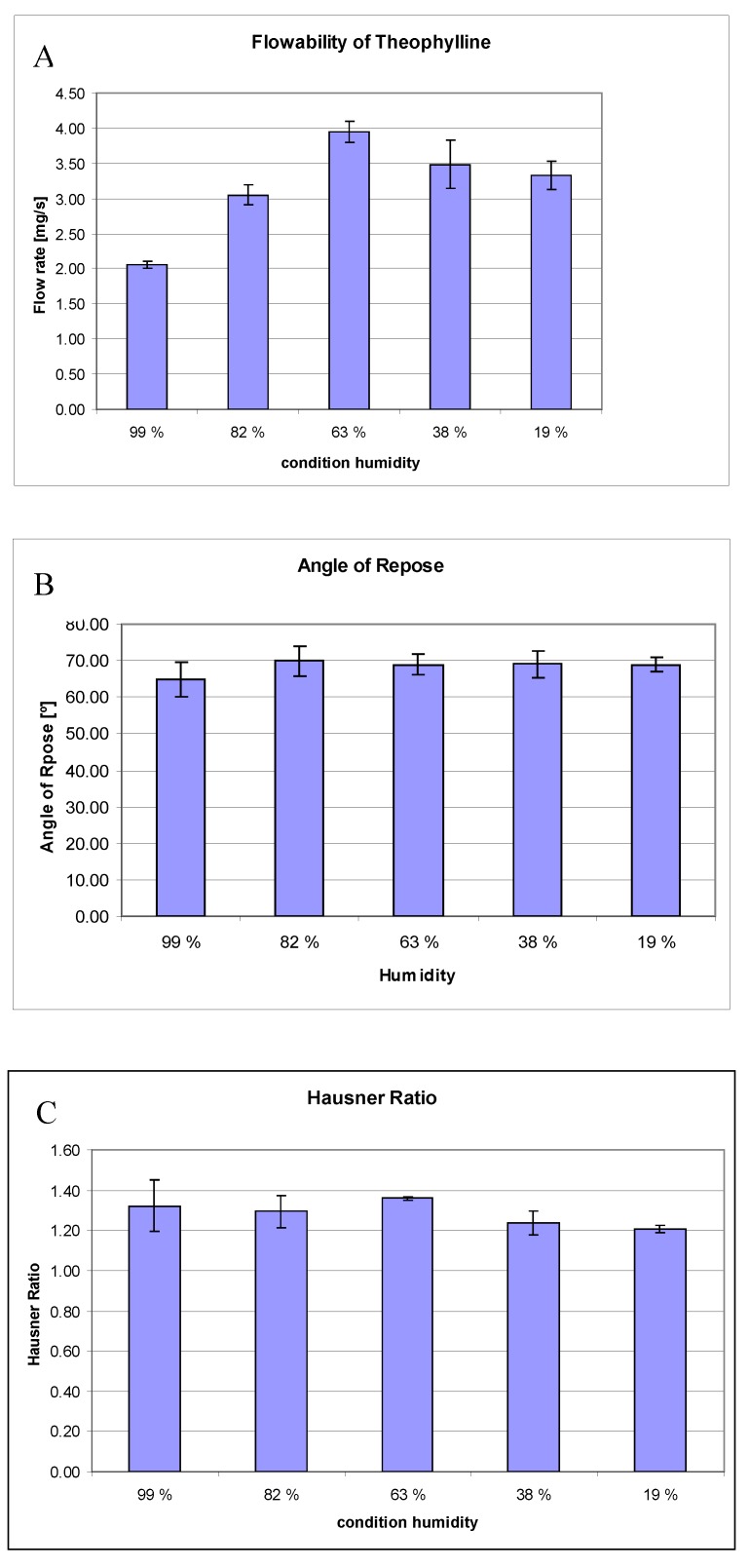
Effects of humidity conditions (RH) on the flow rate of theophylline determined by **(A)** flow rate recording device (FlowPro), **(B)** angle of repose, and **(C)** Hausner ratio (n = 3).

The flow rate of the different conditioned powders is presented in Figure 7. For all measurements, the powder was taken out of the desiccator and sieved directly before the measurement. The differences in powder flowability measured with the FlowPro at different humidities are statistically significant (ANOVA, P-value = 2.71 × 10^-5^). Consequently, the flowability of theophylline powder, as measured with FlowPro, depends on the relative humidity conditions. A pairwise comparison between the different flowabilities shows a statistical significant difference between the 99% RH and the 82% RH conditioned powders (Tukey, P-value = 0.002), as well as between the 82% RH and the 63% RH conditioned samples (Tukey, P-value = 0.007). No significant difference could be proven for the flowability between the 63% RH and the 38% RH conditioned powders (Tukey, P-value = 0.179) and between the 38% RH and the 19% RH conditioned powders (Tukey, P-value = 0.886). The difference in flowability between the 63% RH and the 19% RH conditioned sample is not significant (Tukey, P-value = 0.055). Differences between the angles of repose of theophylline powder after storage at different humidities are not statistically significant (ANOVA, P-value = 0.293). Differences between the Hausner Ratios of theophylline powder after storage at different humidities are not statistically significant (ANOVA, P-value = 0.144). 

As mentioned before, flowability is influenced by powder related factors, such as morphology, size, size distribution, true density, and surface area [11], and chemical composition [5]. These factors were constant for the powders which had been conditioned at 19%, 38%, 63%, and 82% RH. Theophylline that had been conditioned at 99% RH changed its chemical composition and maybe also some other factors. As it has been observed that the smaller particles are coalesced to the surface of the bigger particles, an absolute change in size and size distribution cannot be excluded. Therefore, interpretations for the underlying reason for changes in powder flow properties between the anhydrate and the monohydrate samples have to be made carefully**. ** However, based on the SEM observations, the change in particle and surface morphology is evident in the samples stored at the highest humidity condition studied. This morphological change is likely to have an important influence on the flowability behavior of the powder.

Theophylline showed its best flowability at a relative humidity of 63%. With increasing humidity, the flow rate decreases strongly. The reason for the significant decrease in flowability from the 63% RH conditioned sample to the 82% RH conditioned sample might be that water adsorbs to the surface. Surface water can change the interparticle forces by changing the distance between the particles and finally, the van der Waals forces, and by formation of liquid bridges. The further decrease in flowability for the 99% RH conditioned sample can be explained with the same reasons, but in addition, the change of the chemical composition might influence the flow properties (anhydrate > monohydrate). 

At humidity conditions lower than 63% RH, the flowability of theophylline decreased slightly. Samples conditioned at 38% and 19% RH showed electrostatic repulsion against the steel hopper. Small amounts of water at 63% RH may eliminate electrostatic charges and micro-irregularities, which decrease the flowability at lower humidities. The crystal appearance of theophylline might explain why the effect on the flowability of water bridges at high humidities is stronger than the triboelectic effect at low humidities. As has been summarized in the introduction, crystalline substances adsorb water rather than absorbing it, and consequently, they are of low hygroscopy [15]. The adsorbed water layers lead to the formation of water bridges. Furthermore, relative humidity has a negligible effect on the charging of recipients with low hygroscopicity [27].

The flowability data should be interpreted carefully as the real-time humidity of the powder is not known. Possible mistakes are the adaptation to the circumfluent humidity and changes in humidity while opening the desiccator, due to variation in pressure. Measurements of the bulk and tapped density and the angle of repose were performed slightly later than the FlowPro measurements. The non-significant differences observed between the samples of these classical methods is most likely explained by the poor ability of these classical flow tests to make differences between samples. Based on this study, it would appear that the FlowPro is capable of categorizing dominantly cohesive powder samples with only small differences in their surface properties.

## 4. Conclusions

Solid-state analysis provided insight into the properties of the theophylline samples studied, and more specifically, to the flow behavior of theophylline stored at different humidity conditions. We conclude that the investigated powder flow measurement technique, FlowPro, is capable of detecting even small differences in powder flow properties, which cannot be detected with conventional methods, like the angle of repose or the bulk and tapped density. Small differences in relative humidity can have a great effect on powder flowability. Therefore, conditioning the powder at a humidity close to the flow measuring humidity is necessary. 
